# Agomelatine prevents macrophage infiltration and brain endothelial cell damage in a stroke mouse model

**DOI:** 10.18632/aging.202836

**Published:** 2021-04-04

**Authors:** Yiqiang Cao, Fei Wang, Yonggang Wang, Jiang Long

**Affiliations:** 1 Department of Neurosurgery, The First Affiliated Hospital of Kunming Medical University, Kunming 650032, China

**Keywords:** stroke, ischemic/reperfusions, macrophage, agomelatine, endothelial cells

## Abstract

Background and purpose: Ischemic/reperfusions are regarded as the clinical consensus for stroke treatment, which results in secondary injury of brain tissues. Increased blood-brain barrier (BBB) permeability and infiltration of inflammatory cells are responsible for the ischemic/reperfusion injury. In the present study, we aimed to investigate the effects of Agomelatine on brain ischemic/reperfusions injury and the underlying mechanism.

Methods: MCAO model was established in mice. The expressions of CD68 and claudin-5 in the cerebral cortex were determined using an immunofluorescence assay. Brain permeability was evaluated using Evans blue staining assay. A two-chamber and two-cell trans-well assay was used to detect the migration ability of macrophages through endothelial cells. The expression levels of claudin-5 and MCP-1 in the endothelial cells were determined using qRT-PCR and ELISA.

Results: CD68 was found to be up-regulated in the cerebral cortex of MCAO mice but was down-regulated by treatment with Agomelatine. The expression level of down-regulated claudin-5 in the cerebral cortex of MCAO mice was significantly suppressed by Agomelatine. Deeper staining of Evans blue was found in the MCAO group, which was however faded significantly in the Agomelatine treated MCAO mice. The migrated macrophages were significantly increased by hypoxia incubation but were greatly suppressed by the introduction of Agomelatine. The down-regulated claudin-5 by hypoxic incubation in endothelial cells was up-regulated by treatment with Agomelatine. Furthermore, the increased expression of MCP-1 in endothelial cells under hypoxic conditions was significantly inhibited by Agomelatine.

Conclusion: Agomelatine prevents macrophage infiltration and brain endothelial cell damage in a stroke mouse model.

## INTRODUCTION

Stroke is defined as a syndrome of neurological deficits induced by local or holistic ischemia and hypoxia of brain tissue resulting from an obstruction in cerebrovascular circulation [1, 2]. Focal neurological deficits are reported as the main characteristics of stroke, the clinical symptoms of which include sudden inactiveness on one side of the body, language disorder, and tactus disorders, such as impaired vision and hearing, dizziness, ear irritation, and swallowing difficulty [3]. Reports state that stroke is the secondary inducer of dementia-related brain injury and death amongst the elderly population with more than 60 years of age. The cost of treating stroke takes 3–7% of the overall medical expenditure in developed countries [4]. In China, the mortality of cardiovascular disease has remained in the top three for many years and increases with aging [5]. Stroke adversely affects the quality of life of patients and is also burdensome to the patients' families and society at large. It is of great significance to investigate the pathological mechanism of stroke and explore the potential therapeutic treatments. Ischemic/reperfusion was found to be harmful to the recovery of the tissues when people noticed that a new type of tissue injury could arise from the sudden oxygen supply post-long-term hypoxia, which is also verified in the aggravation of brain dysfunction after ischemic/reperfusion on brain tissues [6, 7]. However, currently, quick recovery of the blood supply has been regarded as the clinical consensus for the treatment of stroke, in which way, ischemic/reperfusion is inevitable. Therefore, the injury to brain tissues resulting from ischemic/reperfusion has been taken as a tough task post clinical stroke treatment.

It is reported that the permeability of the BBB is promoted greatly after the ischemic/reperfusion treatments for stroke, the mechanism of which is currently unknown [8]. With the increase in BBB permeability, inflammation is induced by chemical mediators and infiltration of inflammatory cells, such as macrophages, which further enhances the permeability of the BBB [9]. The BBB is a tight structure that mainly consists of blood endothelial cells, astrocytes, and extracellular matrix [10]. The BBB is connected by tight junction proteins on the endothelial cell surface. There are three types of transmembrane tight junction proteins: claudins, occludin, and junctional adhesion molecules. At the BBB, claudin-5 is the most highly expressed tight junction protein and its dysfunction has been implicated in stroke and brain injury [11]. In the early stage of permeability increase, a widened endothelial gap could be observed instead of any obvious injury. However, as the permeation of the BBB proceeded, inflammatory and chemotactic factors were produced in quantity by astrocytes or infiltrated macrophages around endothelial cells and vessels [12]. One of the key chemotactic factors is MCP-1, and the high levels of MCP-1 have been found in the brain several hours after cerebral ischemia [13]. The expressions of adhesion molecules in endothelial cells are up-regulated by chemical mediators, such as interleukins and tumor necrosis factors. To recruit the leukocytes to the injured location, the metalloprotease (MMP) is activated, by which the ligandin and extracellular matrix are degraded in endothelial cells [14, 15]. The pathological state of brain tissues is further aggravated by up-regulated inflammatory factors, chemotactic factors, MMP, and adhesion molecules [16, 17]. Preventing the infiltration of inflammatory cells, such as macrophages, down-regulating the expressions of chemotactic factors may be an effective way to decrease the permeability of the BBB and protect the brain from the injury by ischemic/reperfusion treatments.

Agomelatine is the first line medication for the treatment of depression. Agomelatine's antidepressant actions attribute to its sleep-promoting and modulation of circadian rhythm by activating melatonin MT1 and MT2 receptors in the suprachiasmatic nucleus and its blockage of 5-HT2c receptors in the hippocampus, amygdale, and prefrontal cortex [18]. Agomelatine treatment inhibits melatonin synthesis, promotes dopamine production in the prefrontal lobe, accelerates epinephrine release in the nucleus ceruleus, and promotes neuron growth [19, 20]. Besides its action on the CNS, Agomelatine exhibits broad effects in other tissues. It has been proposed that the melatonin receptor agonists could possess cardiovascular benefit [21, 22]. Recent studies further reveal the potential effects of their modulation in the cardiovascular system. The experiment *in vitro* shows that Agomelatine alleviates oxidative stress by inhibiting ROS production and promoting antioxidant levels [23]. In a preclinical animal experiment, Agomelatine administration protected animals from lipopolysaccharide-induced cardiovascular toxicity by suppressing the NF-kβ pathway [24]. An anti-depression study found that Agomelatine inhibits the infiltration and polarization of macrophages [25]. Agomelatine therapy increases circulated brain-derived neurotrophic factor BDNF levels but reduces circulating CRP levels in depressive patients [26, 27]. The evidence indicates that the anti-depression drug Agomelatine possesses anti-ROS and anti-inflammatory properties, and it is a highly interesting topic to evaluate the potential therapeutic effect in the cardiovascular disease model. In the present study, the anti-inflammatory effects of Agomelatine on endothelial cells were investigated to explore its potential therapeutic effects on injury induced by ischemic/reperfusion treatments for stroke.

## RESULTS

### Agomelatine inhibited macrophage infiltration in the cortex of the MCAO mouse model

**Figure 1:**
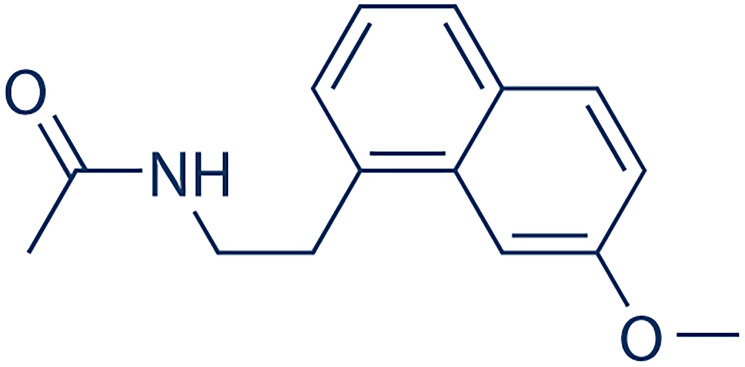
Molecular structure of Agomelatine.

**Figure 2:**
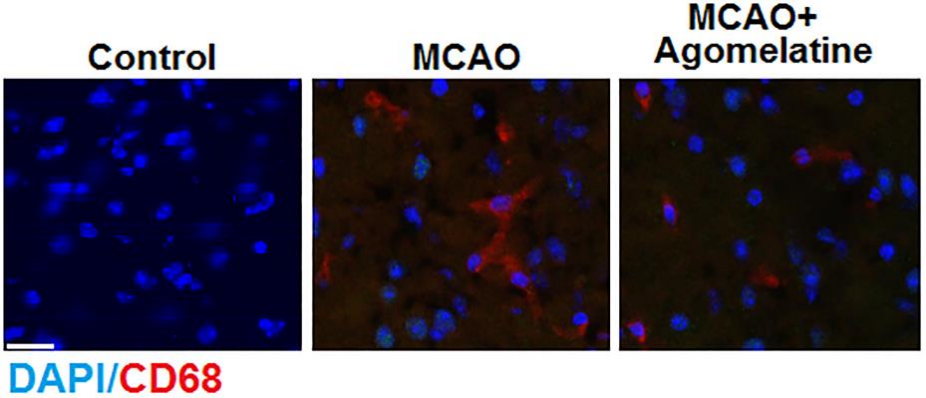
**Agomelatine prevents macrophage infiltration in the cortex of the MCAO mouse model.** Representative images of staining for macrophages using CD68 in the cerebral cortex 3 days post-tMCAO. Scale bar, 100 μM.

To evaluate the effects of Agomelatine on the infiltration of macrophages in MCAO mice, the MCAO mice were treated with Agomelatine and the cortex was isolated to determine the expression of CD68 by immunofluorescence, which is a representative marker of M2 macrophages. The structure of Agomelatine is shown in Figure 1. As shown in Figure 2, CD68 was found to be up-regulated in the cortex isolated from MCAO mice, compared to the control, which was significantly reversed by the treatment with Agomelatine. These data indicate that the infiltration of macrophages into the cortex was greatly increased by establishing the MCAO model in mice and was alleviated by Agomelatine.

### Agomelatine promoted the expression of the down-regulated tight junction protein in the MCAO mouse model

**Figure 3:**
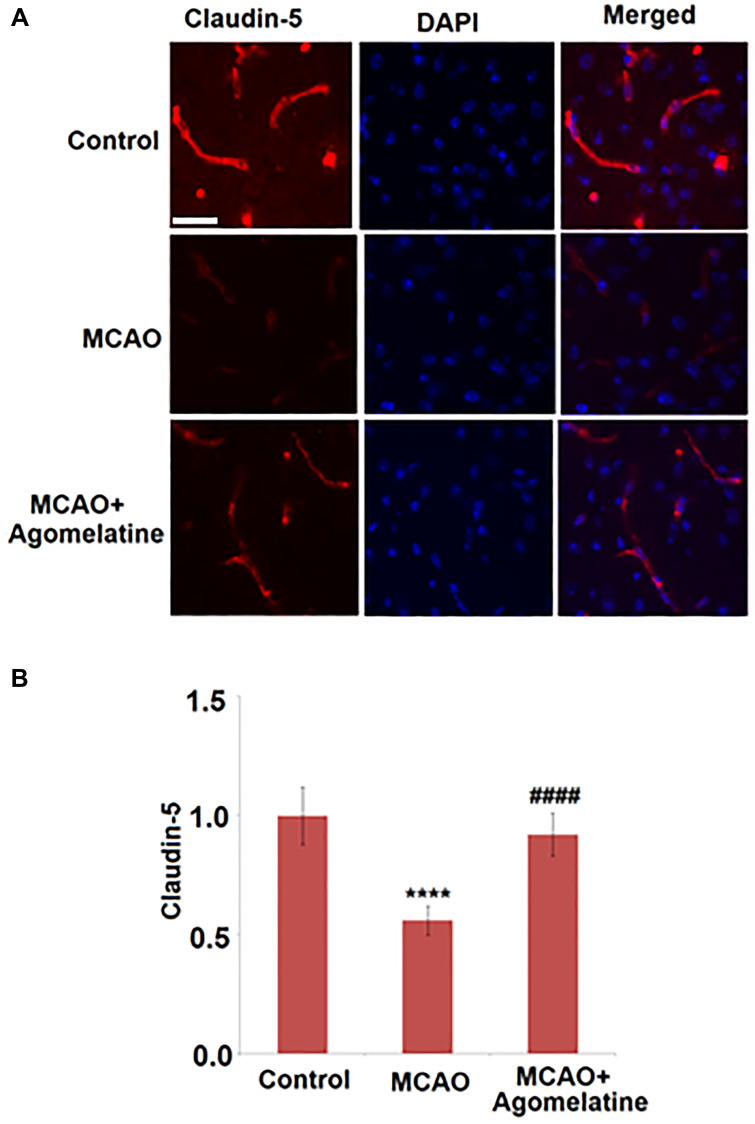
**Agomelatine restored the reduction of tight junction protein expressions in the MCAO mouse model.** (**A**). Representative images of staining for claudin-5 in the cerebral cortex 3 days post-tMCAO; (**B**). Quantification of claudin-5 staining. Scale bar, 100 μM (^****^P < 0.0001 vs. vehicle group; ^####^*P* < 0.0001 vs. MCAO group).

To explore the effects of Agomelatine on the tight junctions between endothelial cells in MCAO mice, the expression level of claudin-5 was evaluated. As shown in Figure 3, claudin-5 was significantly down-regulated in the cortex by MCAO modeling in mice but was greatly up-regulated by the introduction of Agomelatine, indicating that the damaged tight junction by MCAO modeling was repaired by Agomelatine.

### BBB permeability in MCAO mice was diminished by Agomelatine

**Figure 4:**
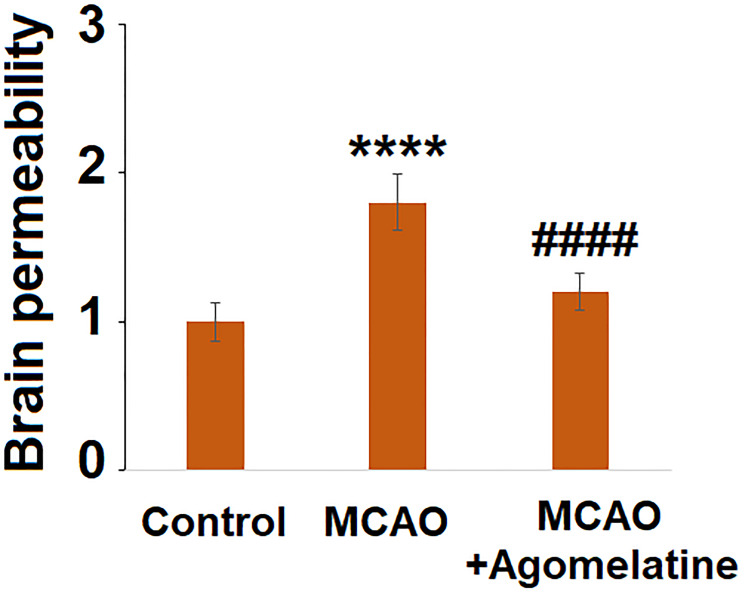
**Agomelatine protects against impairment of brain permeability in MCAO mice.** Brain permeability of MCAO mice was measured by Evans blue staining assay (^****^P < 0.0001 vs. vehicle group; ^####^*P* < 0.0001 vs. MCAO group).

The permeability of the BBB is of great importance in preventing damage to brain tissues by chemical materials and infiltrated inflammatory cells. Following MCAO modeling and Agomelatine administration, the permeability of the BBB of each animal was evaluated by the Evans blue staining assay. As shown in Figure 4, deeper staining of Evans blue was found in the MCAO group which was however faded significantly in Agomelatine treated MCAO animals. These data indicate that the increased BBB permeability by MCAO modeling was reversed by Agomelatine.

### The migration of macrophages was suppressed by Agomelatine in hypoxic endothelial cells

**Figure 5:**
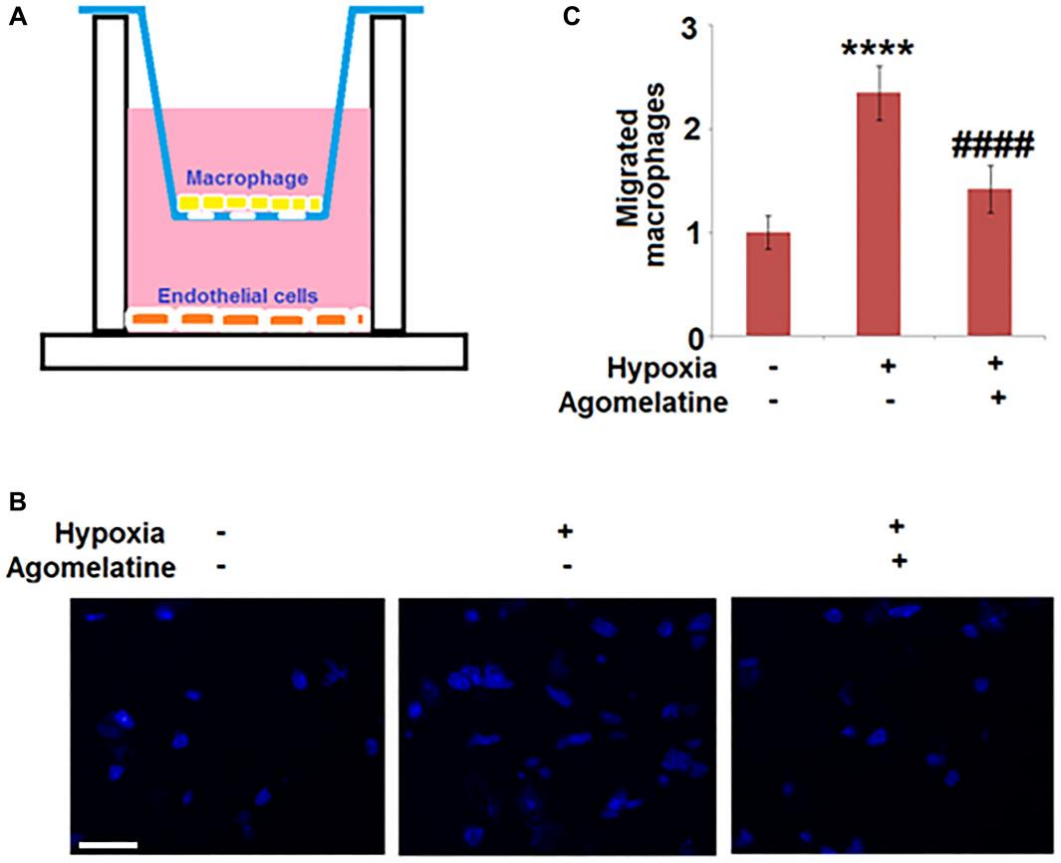
**Agomelatine treated hypoxic endothelial cells inhibit macrophage migration.** Brain bEnd.3 endothelial cells were incubated with 10 μM Agomelatine in the process of hypoxia/ reperfusion. (**A**). A representative schematic of the two-chamber two-cell trans-well assay. Brain bEnd.3 endothelial cells were subjected to hypoxia/reoxygenation (H/R) are plated in the bottom chamber and macrophages (IC21) are suspended in the top chamber and allowed to migrate. (**B**). Migrated IC21 macrophages were stained with DAPI; (**C**). Quantification of migrated macrophages. Scale bar, 100 μM (^****^P < 0.0001 vs. vehicle group; ^####^*P* < 0.0001 vs. H/R group).

Following hypoxic incubation, bEnd.3 cells were incubated with 10 μM Agomelatine. A two-chamber two-cell trans-well assay was performed to test the migration ability of macrophages through bEnd.3 cells. Figure 5A shows the representative schematic of the assay. BEnd.3 endothelial cells were plated in the bottom chamber and macrophages (IC21 cells) were suspended in the top chamber and allowed to migrate through the endothelial cells layer. As shown in Figure 5B and 5C, compared to the control, the number of migrated macrophages was significantly increased by hypoxia incubation, but was greatly suppressed by the introduction of Agomelatine, indicating that hypoxia incubation-induced macrophage migration was inhibited by Agomelatine.

### The expression level of claudin-5 was up-regulated by Agomelatine in hypoxic endothelial cells

**Figure 6:**
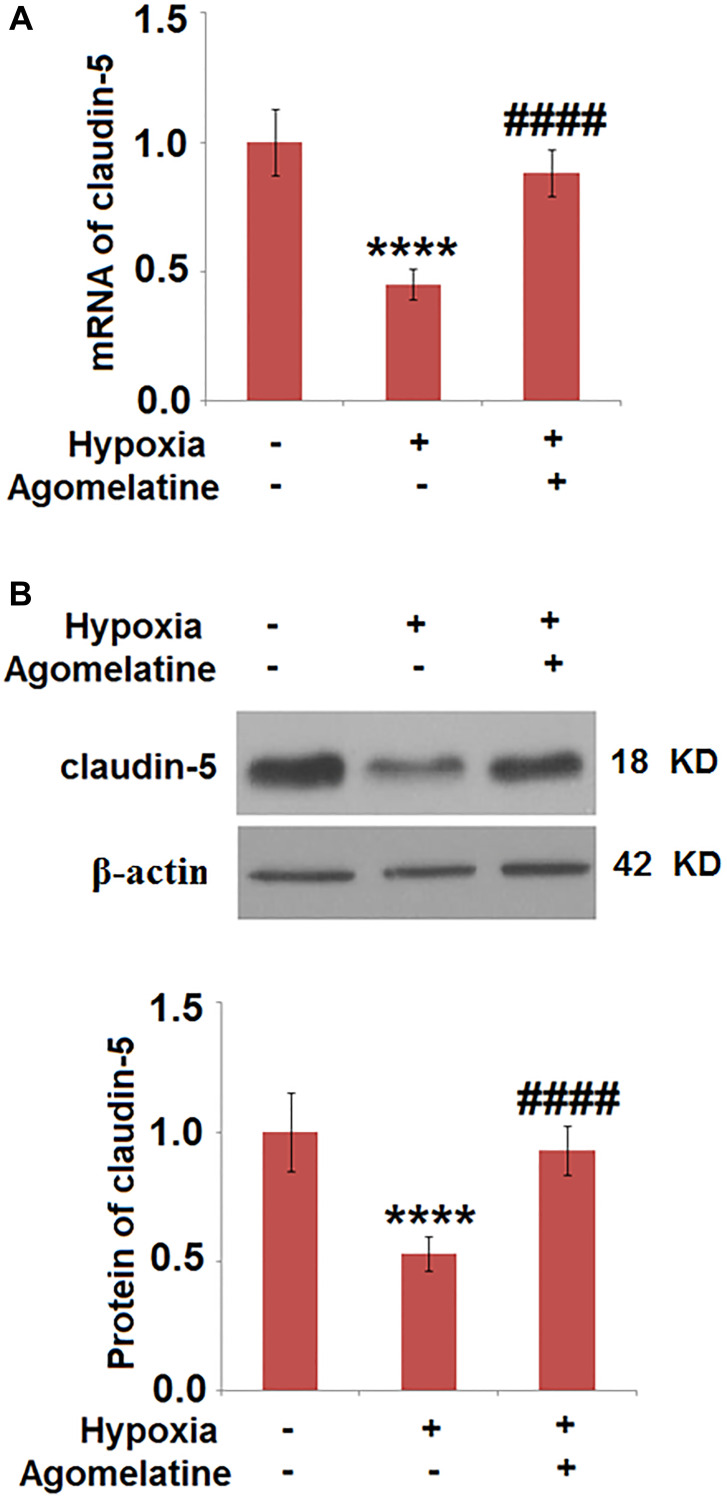
**Agomelatine restored the expression of claudin-5 in hypoxic endothelial cells.** Brain bEnd.3 endothelial cells were incubated with 10 μM Agomelatine in the process of hypoxia/ reperfusion (H/R). (**A**). mRNA of claudin-5 as measured by real-time PCR; (**B**). Protein expression of claudin-5 as measured by western blot analysis (^****^P < 0.0001 vs. vehicle group; ^####^*P* < 0.0001 vs. H/R group).

To evaluate the effects of Agomelatine on the tight junctions between hypoxic endothelial cells, the expression level of claudin-5 was evaluated by qRT-PCR and western blot analysis. As shown in Figure 6, claudin-5 was significantly down-regulated by hypoxic incubation, the expression of which was greatly restored by Agomelatine. These data indicate that the injured tight junctions between endothelial cells by hypoxic incubation were significantly repaired by Agomelatine.

### MCP-1 was down-regulated by Agomelatine in hypoxic endothelial cells

**Figure 7:**
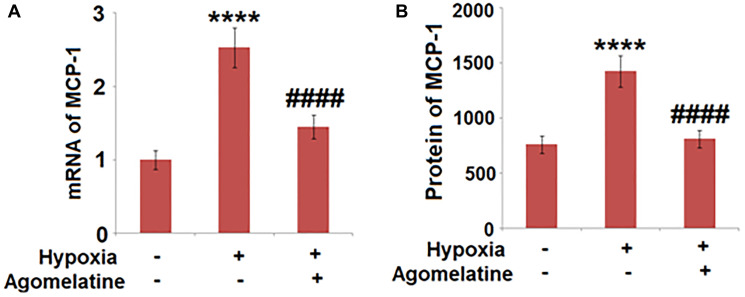
**Agomelatine reduced the expression and secretion of MCP-1 in hypoxic endothelial cells.** Brain bEnd.3 endothelial cells were incubated with 10 μM Agomelatine in the process of hypoxia/ reperfusion (H/R). (**A**). mRNA of MCP-1 was measured; (**B**). Secretions of MCP-1 were measured (^****^P < 0.0001 vs. vehicle group; ^####^*P* < 0.0001 vs. H/R group).

To explore the effects of Agomelatine on the production of chemotactic factors, the expression of MCP-1, a representative chemotactic factor, was determined in the hypoxic endothelial cells in the presence or absence of Agomelatine. As shown in Figure 7, MCP-1 was greatly up-regulated by hypoxic incubation, the expression of which was significantly decreased by Agomelatine, indicating that the chemotactic function of endothelial cells to inflammatory cells, such as macrophages, was suppressed by Agomelatine.

## DISCUSSION

The brain is the most sensitive organ to hypoxia in mammals and vital activity within the brain is mainly dependent on the energy provided by the aerobic oxidation of glucose. Therefore, irreversible injury is induced under a long-time hypoxic environment [28, 29]. Thrombolytic therapy and mechanical recanalization are effective methods for blood re-supply after ischemia. However, for some specific patients, the injury induced by ischemia can be aggravated by ischemic/reperfusion, which is termed as “brain ischemic/reperfusion injury” [30].

Multiple types of pathological processes are involved in the injury, including changes in energy and amino acid metabolism, the cellular overload of calcium, activation of reactive oxygen species, inflammatory reactions, and damage of the BBB, which culminates in the apoptosis of neurons [31, 32]. Ischemic/reperfusion is usually accompanied by the overexpression of inflammatory factors and adhesion molecules, as well as the infiltration of leukocytes. Direct damage is induced on the BBB by proteolytic enzymes produced by infiltrated leukocytes, including MMP, reactive oxygen species, and the metabolites of arachidonic acid [33, 34]. As verified by the clinical trials and animal experiments, brain ischemic/reperfusion injury can be induced by vasogenic cerebral edema, hemorrhagic transformation, and infarction resulting from the destruction of the BBB [35]. In the present study, a middle cerebral artery occlusion (MCAO) model was established in mice to evaluate the therapeutic effects of Agomelatine on brain ischemic/reperfusion. We found that the permeability of the BBB was significantly increased by MCAO modeling, which was confirmed by the results of Evans blue staining and repaired by the treatment with Agomelatine, indicating recovery effects of Agomelatine on by MCAO modeling-induced BBB injury. The repair function of Agomelatine on the BBB was indirectly claimed by its promising inhibitory effects on the infiltration of macrophages into the brain tissue. We found that the increased infiltrated macrophages induced by MCAO modeling, especially M2 macrophages, were significantly suppressed by the introduction of Agomelatine. In *in vitro* macrophage migration assay, macrophages were found to easily migrate through endothelial cells under hypoxic conditions compared to endothelial cells under regular conditions or cells under hypoxic conditions but incubated with Agomelatine, indicating the promising inhibitory effects of Agomelatine on macrophage migration. We suspect that the inhibitory effects of Agomelatine on macrophage migration or infiltration are related to its repair function on the tight junctions between endothelial cells, which are the main components of the BBB. To verify our hypothesis, the expression level of a representative protein of the tight junctions between endothelial cells, claudin-5, was evaluated both *in-vivo* and *in vitro*. We found that in both the cerebral cortex of MCAO mice and the endothelial cells under hypoxic conditions, claudin-5 was significantly down-regulated, indicating destructed tight junctions. As predicted, the decreased expression of claudin-5 was promoted by Agomelatine, claiming a potential mechanism underlying the effects of Agomelatine on macrophage infiltration and BBB permeability by enhancing the tight junctions between endothelial cells.

Integrated inflammatory reactions are initiated by the central nervous system to prevent the brain from external injuries and acute inflammatory reactions play an important role in the secondary injury induced by brain ischemic/reperfusion. Following ischemic/reperfusion, the production of adhesion molecules and chemotactic factors are promoted to recruit more and more leukocytes, macrophages, and neutrophil granulocytes [36]. The activated and recruited macrophages and neutrophil granulocytes migrate and adhere to the endothelial cells, which is reported to be initiated 1-hour post-ischemic/reperfusion and achieves the peak at approximately 24–48 hours post-ischemic/reperfusion. Multiple types of pro-inflammatory mediators, such as reactive oxygen species, proteases, and lysosomal enzymes, are released by the infiltrated macrophages and neutrophil granulocytes, which induce the secondary injury on brain tissue post- ischemic/reperfusion. Leukocytes trigger injury to capillaries by changing the hemorheology, diameter. and permeability of capillaries [37, 38]. In the present study, the expression level of MCP-1, a representative chemotactic factor, was evaluated to claim the effects of Agomelatine on the chemotactic function of injured endothelial cells to inflammatory cells. We found that MCP-1 was significantly up-regulated by hypoxia and down-regulated by Agomelatine, indicating a potential chemotactic inhibitory effect of Agomelatine under the secondary injury of ischemic/reperfusion.

There were limitations to the study, one of which is that we still do not fully understand the protective mechanism of Agomelatine acting on brain endothelial cells and macrophages in stroke animals. The effect of Agomelatine could be dependent on its suppression of 5-HT2C or its activation of melatonin receptors. The role of 5-HT2c in ischemic injury has not been well confirmed [39]. Melatonin MT1 and MT2 receptors have been shown to be involved in ischemic diseases. Activation of the MT1 receptor inhibits neuronal mitochondrial cell death pathways, while the action of the MT2 receptor protects against ischemia/reperfusion injury [40]. In isolated brain endothelial cells, MT1 and MT2 are required for protecting the BBB from neurotoxin-induced impairment [41]. Macrophages expressing MT1 and MT2 are reported to regulate macrophage polarization involving multiple pathways including NF-κB [42] Therefore, the MT receptor-mediated pathway could be an important mechanism that Agomelatine protects against brain injury following stroke associated with ischemia/reperfusion. In addition, since Agomelatine has been shown to exhibit pleiotropic effects, some of the mechanism acting on neuroprotection could be independent of its binding to melatonin receptors or 5-HT2c. A recent study shows a short time injection of Agomelatine inhibits myocardial mitochondrial permeability transition pore opening and improves myocardial ischemia injury in an isolated rat heart model [43]. It is likely that Agomelatine could also protect the neuronal mitochondria from ischemia conditioning. Further investigation should be performed on the underlying molecular mechanism to illustrate more clearly how Agomelatine exerts neuroprotection in our future study.

## MATERIALS AND METHODS

Collectively, we found that Agomelatine might prevent macrophage infiltration and brain endothelial cell damage in a stroke mouse model.

### Middle cerebral artery occlusion (MCAO) model and drug administration

Twelve CD-1 mice were housed in a 12 hour light/dark cycle, temperature-controlled room, and divided into 3 groups randomly. Agomelatine (10 mg/kg/day, Servier Laboratories, Suresnes, France) or its vehicle HEC (hydroxyethyl cellulose 1%, Servier Laboratories) were administered intraperitoneally (i.p.) on a daily basis for 15 days [44, 45]. Animals were used to establish the MCAO model according to the procedure reported by Kusaka [46]. Briefly, mice were anesthetized intraperitoneally with a mixture of ketamine (80 mg/kg) and xylazine (20 mg/kg). Body temperatures and respiration rates were recorded perioperatively. A vertical midline cervical incision was conducted, and the animals' right common carotid artery was exposed. The external carotid artery was isolated, coagulated, transected, and divided (leaving 3–4 mm). The internal carotid artery was isolated, and the pterygopalatine artery was ligated close to its origin. A 5 mm aneurysm clip was utilized to fix the common carotid artery. The external carotid artery stump was reopened, and a 4.0 monofilament nylon suture was inserted through the internal carotid artery. The insertion was stopped when resistance was felt, occluding the origin of the right MCA. Post 2 hours of occlusion, the suture was withdrawn to allow for reperfusion. The external carotid artery was ligated, and the aneurysm clip was removed. The skin was sutured (1% lidocaine was applied), and the animal was left to recover. 98% purity of Agomelatine was purchased from Sigma (St. Louis, MO).

### Immunofluorescence

The forebrain tissues were collected and sectioned using a Cryostats. The cortex tissue was then fixed using 100% cold methanol for 10 minutes and permeabilized with 0.1% Triton-X in PBS. The slides were then blocked and incubated with primary rabbit anti-CD68 or anti-claudin-5 (1:1000, Abcam, Cambridge, MA) antibody overnight at 4°C. Following three washes with PBS, cells were incubated with secondary Cy3-conjugated anti-rabbit IgG (1:200, Abcam, Cambridge, MA) for an additional 30 min at room temperature. DAPI was added to dye the nucleus for 5 minutes and 50% glycerinum was used to block the medium. Stained cells were photographed under a fluorescence microscope (Olympus, Tokyo, Japan).

### Evans Blue (EB) extravasation assay

To examine BBB integrity, EB extravasation assays were performed. Following drug treatment and the MCAO experiment, mice were injected with 0.25 mL EB dye (2%) (Sigma, St. Louis, MO) via the caudal vein. Two hours later, the animals were anaesthetized and killed by saline infusion. The left hemisphere of each brain was weighed, added into 500 μL N, N-dimethylformamide, and incubated at 72°C for 3 days. Samples were centrifuged twice at 1516 g for 20 minutes. The supernatant was collected and aliquoted (200 μL) into a 96-well glass plate. Fluorescence was quantified using a spectrophotometer at an excitation wavelength of 620 nm and an emission wavelength of 680 nm.

### Cell culture and hypoxic injury model

The mouse microvascular endothelial cell line, bEnd.3 cells, were purchased from American Type Culture Collection (ATCC, Rockville, MD USA). DMEM medium with 10% fetal bovine serum was used to culture the cells at 37°C with 5% CO_2_. The hypoxia model was established according to a previously reported protocol [47]. Cells were placed in a hypoxia incubator (1% O_2_, 5% CO_2_, 94% N_2_) for 8 hours. An automated regulator with a built-in flow meter and oxygen sensor was used to ensure and maintain the proper composition of gas mixture within the incubator. After hypoxia treatment, cells were removed from the chamber and utilized for the subsequent experiments.

### Trans-well migration assay

An approximate 5 × 10^5^ macrophages were suspended in serum-free DMEM and plated into the upper insert of a six-well trans-well plate (BD, Minneapolis, MN) and 5 × 10^5^ bEnd.3 cells were plated into the bottom of the lower chamber. The cells were incubated at 37°C for 8 hours. The non-migratory cells in the upper layer were removed and the migratory cells were fixed with 4% paraformaldehyde at room temperature for 10 minutes, followed by staining with DAPI. Images were photographed and quantified by counting cell numbers of five randomly picked fields of view for each well.

### Reverse transcriptase-polymerase chain reaction (qRT-PCR)

Total RNA was collected from the cells using an RNA Extraction Kit (Thermo Fisher Scientific, Waltham, USA) in terms of the instructions of the manufacture. Extracted RNA was quantified with a NanoDrop spectrophotometer (Thermo Fisher Scientific, Waltham, USA). A specific RT primer was used to reverse-transcribe the complementary DNA. SYBR Premix Ex Taq TM (Thermo Fisher Scientific, Waltham, USA) with an Applied Bio-Rad CFX96 Sequence Detection system (Genscript, NanJing, China) was used in the real-time PCR procedure. The following primers were used in this study: claudin-5 (Forward: 5′- ACGGGAGGAGCGCTTTAC -3′, Reverse: 5′- GTTGGCGAACCAGCAGAG -3′); MCP-1: (Forward: 5′- CCCAGGAGTGCCTTGATTC -3′, Reverse: 5′-CGCCCCATAATTCTGACATC -3′); GAPDH (Forward: 5′- AAGAGGGATGCTGCCCTTAC -3′, Reverse: 5′- CCATTTTGTCTACGGGACGA-3′). The expression levels of claudin-5 and MCP-1 were determined by the threshold cycle (Ct), and relative expression levels were calculated using the 2^−ΔΔCt^ method.

The expression level of GAPDH in the tissue was taken as a negative control.

### Western blot assay

Total proteins were isolated from cells using the nuclear and cytoplasmic protein extraction kit (Thermo Fisher Scientific, Waltham, USA). Approximately 40 μg of the protein were separated on 12% SDS-polyacrylamide gel (SDS-PAGE) and the gel was transferred to a polyvinylidene difluoride (PVDF) membrane (Millipore, MIT, USA). The membrane was blocked with 5% non-fat dry milk in TBST (Trisbuffered saline/0.1% Tween-20, pH 7.4) for 1 hour at room temperature and incubated overnight with primary antibodies against claudin-5 (1:1000), MCP-1 (1:1000) and GAPDH (1:1000) (Abcam, Cambridge, MA). A horseradish peroxidase (HRP)-conjugated antibody against rabbit IgG (1:5000, Abcam, Cambridge, MA) was used as a secondary antibody. Blots were incubated with the ECL reagents (Amersham Pharmacia Biotech, Inc, USA) and exposed to Tanon 5200-multi to detect protein expression. The intensity of bands was quantified using Image Lab software (Bio-Rad, Herculus, USA). The relative expression of the protein was normalized to the control sample and presented as fold change to the initial value.

### Statistical analysis

Mean ± standard deviation (SD) was displayed to show the data. GraphPad Prism 6.0 (GraphPad, China) was used to analyze the data. Analysis of variance (ANOVA) followed by Tukey’s HSD post-hoc test was utilized for the contrast among different groups. *P* < 0.05 was regarded as a statistically significant difference between the groups.

## References

[1] Charriaut-Marlangue C, Besson VC, Baud O. Sexually Dimorphic Outcomes after Neonatal Stroke and Hypoxia-Ischemia. Int J Mol Sci. 2017; 19:61. 10.3390/ijms19010061 29278365 PMC5796011

[2] Nathaniel TI, Williams-Hernandez A, Hunter AL, Liddy C, Peffley DM, Umesiri FE, Imeh-Nathaniel A. Tissue hypoxia during ischemic stroke: adaptive clues from hypoxia-tolerant animal models. Brain Res Bull. 2015; 114:1–12. 10.1016/j.brainresbull.2015.02.006 25738761

[3] Baldassarre A, Ramsey LE, Siegel JS, Shulman GL, Corbetta M. Brain connectivity and neurological disorders after stroke. Curr Opin Neurol. 2016; 29:706–13. 10.1097/WCO.0000000000000396 27749394 PMC5682022

[4] Chamorro Á, Dirnagl U, Urra X, Planas AM. Neuroprotection in acute stroke: targeting excitotoxicity, oxidative and nitrosative stress, and inflammation. Lancet Neurol. 2016; 15:869–81. 10.1016/S1474-4422(16)00114-9 27180033

[5] Feigin VL, Forouzanfar MH, Krishnamurthi R, Mensah GA, Connor M, Bennett DA, Moran AE, Sacco RL, Anderson L, Truelsen T, O'Donnell M, Venketasubramanian N, Barker-Collo S, et al. Global and regional burden of stroke during 1990-2010: findings from the Global Burden of Disease Study 2010. Lancet. 2014; 383:245–54. 10.1016/s0140-6736(13)61953-4 24449944 PMC4181600

[6] Hearse DJ, Humphrey SM, Chain EB. Abrupt reoxygenation of the anoxic potassium-arrested perfused rat heart: a study of myocardial enzyme release. J Mol Cell Cardiol. 1973; 5:395–407. 10.1016/0022-2828(73)90030-8 4355339

[7] Reimer KA, Lowe JE, Rasmussen MM, Jennings RB. The wavefront phenomenon of ischemic cell death. 1. Myocardial infarct size vs duration of coronary occlusion in dogs. Circulation. 1977; 56:786–94. 10.1161/01.cir.56.5.786 912839

[8] Khatri R, McKinney AM, Swenson B, Janardhan V. Blood-brain barrier, reperfusion injury, and hemorrhagic transformation in acute ischemic stroke. Neurology. 2012; 79:S52–57. 10.1212/WNL.0b013e3182697e70 23008413

[9] Payen JF, Fauvage B, Falcon D, Lavagne P. [Brain oedema following blood-brain barrier disruption: mechanisms and diagnosis]. Ann Fr Anesth Reanim. 2003; 22:220–25. 10.1016/s0750-7658(03)00010-8 12747990

[10] Neuwelt EA, Bauer B, Fahlke C, Fricker G, Iadecola C, Janigro D, Leybaert L, Molnár Z, O'Donnell ME, Povlishock JT, Saunders NR, Sharp F, Stanimirovic D, et al. Engaging neuroscience to advance translational research in brain barrier biology. Nat Rev Neurosci. 2011; 12:169–82. 10.1038/nrn2995 21331083 PMC3335275

[11] Greene C, Hanley N, Campbell M. Claudin-5: gatekeeper of neurological function. Fluids Barriers CNS. 2019; 16:3. 10.1186/s12987-019-0123-z 30691500 PMC6350359

[12] Amantea D, Micieli G, Tassorelli C, Cuartero MI, Ballesteros I, Certo M, Moro MA, Lizasoain I, Bagetta G. Rational modulation of the innate immune system for neuroprotection in ischemic stroke. Front Neurosci. 2015; 9:147. 10.3389/fnins.2015.00147 25972779 PMC4413676

[13] Wang X, Yue TL, Barone FC, Feuerstein GZ. Monocyte chemoattractant protein-1 messenger RNA expression in rat ischemic cortex. Stroke. 1995; 26:661–65. 10.1161/01.str.26.4.661 7709415

[14] Liu J, Jin X, Liu KJ, Liu WL. Matrix metalloproteinase-2-mediated occludin degradation and caveolin-1-mediated claudin-5 redistribution contribute to blood-brain barrier damage in early ischemic stroke stage. J Neurosci. 2012; 32:3044–57. 10.1523/JNEUROSCI.6409-11.2012 22378877 PMC3339570

[15] Asahi M, Asahi K, Jung JC, del Zoppo GJ, Fini ME, Lo EH. Role for matrix metalloproteinase 9 after focal cerebral ischemia: effects of gene knockout and enzyme inhibition with BB-94. J Cereb Blood Flow Metab. 2000; 20:1681–89. 10.1097/00004647-200012000-00007 11129784

[16] Dimitrijevic OB, Stamatovic SM, Keep RF, Andjelkovic AV. Effects of the chemokine CCL2 on blood-brain barrier permeability during ischemia-reperfusion injury. J Cereb Blood Flow Metab. 2006; 26:797–810. 10.1038/sj.jcbfm.9600229 16192992

[17] del Zoppo GJ. Inflammation and the neurovascular unit in the setting of focal cerebral ischemia. Neuroscience. 2009; 158:972–82. 10.1016/j.neuroscience.2008.08.028 18824084 PMC2665879

[18] De Berardis D, Fornaro M, Serroni N, Campanella D, Rapini G, Olivieri L, Srinivasan V, Iasevoli F, Tomasetti C, De Bartolomeis A, Valchera A, Perna G, Mazza M, et al. Agomelatine beyond borders: current evidences of its efficacy in disorders other than major depression. Int J Mol Sci. 2015; 16:1111–30. 10.3390/ijms16011111 25569089 PMC4307293

[19] Hale A, Corral RM, Mencacci C, Ruiz JS, Severo CA, Gentil V. Superior antidepressant efficacy results of agomelatine versus fluoxetine in severe MDD patients: a randomized, double-blind study. Int Clin Psychopharmacol. 2010; 25:305–14. 10.1097/YIC.0b013e32833a86aa 20856123

[20] Depreux P, Lesieur D, Mansour HA, Morgan P, Howell HE, Renard P, Caignard DH, Pfeiffer B, Delagrange P, Guardiola B, Yous S, Demarque A, Adam G, et al. Synthesis and structure-activity relationships of novel naphthalenic and bioisosteric related amidic derivatives as melatonin receptor ligands. J Med Chem. 1994; 37:3231–39. 10.1021/jm00046a006 7932550

[21] Paulis L, Simko F, Laudon M. Cardiovascular effects of melatonin receptor agonists. Expert Opin Investig Drugs. 2012; 21:1661–78. 10.1517/13543784.2012.714771 22916799

[22] Laudon M, Frydman-Marom A. Therapeutic effects of melatonin receptor agonists on sleep and comorbid disorders. Int J Mol Sci. 2014; 15:15924–50. 10.3390/ijms150915924 25207602 PMC4200764

[23] Yao K, Zhao YF, Zu HB. Melatonin receptor stimulation by agomelatine prevents Aβ-induced tau phosphorylation and oxidative damage in PC12 cells. Drug Des Devel Ther. 2019; 13:387–96. 10.2147/DDDT.S182684 30718944 PMC6345325

[24] Asci H, Ozmen O, Erzurumlu Y, Sofu A, Icten P, Kaynak M. Agomelatine protects heart and aorta against lipopolysaccharide-induced cardiovascular toxicity via inhibition of NF-kβ phosphorylation. Drug Chem Toxicol. 2019; 13:1–10. 10.1080/01480545.2019.1663209 31514555

[25] Kalkman HO, Feuerbach D. Antidepressant therapies inhibit inflammation and microglial M1-polarization. Pharmacol Ther. 2016; 163:82–93. 10.1016/j.pharmthera.2016.04.001 27101921

[26] Martinotti G, Pettorruso M, De Berardis D, Varasano PA, Lucidi Pressanti G, De Remigis V, Valchera A, Ricci V, Di Nicola M, Janiri L, Biggio G, Di Giannantonio M. Agomelatine Increases BDNF Serum Levels in Depressed Patients in Correlation with the Improvement of Depressive Symptoms. Int J Neuropsychopharmacol. 2016; 19:pyw003. 10.1093/ijnp/pyw003 26775293 PMC4886672

[27] De Berardis D, Fornaro M, Orsolini L, Iasevoli F, Tomasetti C, De Bartolomeis A, Serroni N, De Lauretis I, Girinelli G, Mazza M, Valchera A, Carano A, Vellante F, et al. Effect of agomelatine treatment on C-reactive protein levels in patients with major depressive disorder: an exploratory study in "real-world," everyday clinical practice. CNS Spectr. 2017; 22:342–47. 10.1017/S1092852916000572 27702411

[28] Singh DK, Ling EA, Kaur C. Hypoxia, and myelination deficits in the developing brain. Int J Dev Neurosci. 2018; 70:3–11. 10.1016/j.ijdevneu.2018.06.012 29964158

[29] Ping FC, Jenkins LC. Protection of the brain from hypoxia: a review. Can Anaesth Soc J. 1978; 25:468–73. 10.1007/BF03007408 365304

[30] Chen X, Patra A, Sadowska GB, Stonestreet BS. Ischemic- Reperfusion Injury Increases Matrix Metalloproteinases and Tissue Metalloproteinase Inhibitors in Fetal Sheep Brain. Dev Neurosci. 2018; 40:234–45. 10.1159/000489700 30048980 PMC6557634

[31] Disdier C, Chen X, Kim JE, Threlkeld SW, Stonestreet BS. Anti-Cytokine Therapy to Attenuate Ischemic-Reperfusion Associated Brain Injury in the Perinatal Period. Brain Sci. 2018; 8:101. 10.3390/brainsci8060101 29875342 PMC6025309

[32] Chen XM, Chen HS, Xu MJ, Shen JG. Targeting reactive nitrogen species: a promising therapeutic strategy for cerebral ischemia-reperfusion injury. Acta Pharmacol Sin. 2013; 34:67–77. 10.1038/aps.2012.82 22842734 PMC4086503

[33] Luo C, Yi B, Fan W, Chen K, Gui L, Chen Z, Li L, Feng H, Chi L. Enhanced angiogenesis and astrocyte activation by ecdysterone treatment in a focal cerebral ischemia rat model. Acta Neurochir Suppl. 2011; 110:151–55. 10.1007/978-3-7091-0353-1_26 21116931

[34] Kim JM, Kim S, Kim DH, Lee CH, Park SJ, Jung JW, Ko KH, Cheong JH, Lee SH, Ryu JH. Neuroprotective effect of forsythiaside against transient cerebral global ischemia in gerbil. Eur J Pharmacol. 2011; 660:326–33. 10.1016/j.ejphar.2011.03.051 21501605

[35] Murakami Y, Zhao Q, Harada K, Tohda M, Watanabe H, Matsumoto K. Choto-san, a Kampo formula, improves chronic cerebral hypoperfusion-induced spatial learning deficit via stimulation of muscarinic M1 receptor. Pharmacol Biochem Behav. 2005; 81:616–25. 10.1016/j.pbb.2005.05.004 15936808

[36] Shuaib A. The role of taurine in cerebral ischemia: studies in transient forebrain ischemia and embolic focal ischemia in rodents. Adv Exp Med Biol. 2003; 526:421–31. 10.1007/978-1-4615-0077-3_51 12908627

[37] Sharma S, Gupta S. Neuroprotective effect of MnTMPyP, a superoxide dismutase/catalase mimetic in global cerebral ischemia is mediated through reduction of oxidative stress and DNA fragmentation. Eur J Pharmacol. 2007; 561:72–79. 10.1016/j.ejphar.2006.12.039 17320858

[38] Iqbal S, Baziany A, Hussain M, James S, Wright S, Hemmings S, Shuaib A, Rajput A. Trimetazidine as a potential neuroprotectant in transient global ischemia in gerbils: a behavioral and histological study. Brain Res. 2002; 928:1–7. 10.1016/s0006-8993(01)03095-5 11844466

[39] Torup L, Diemer NH. Is the 5-HT2C receptor a therapeutic target in cerebral ischemia? Pharmacol Toxicol. 2000; 87:74–78. 10989944 10.1034/j.1600-0773.2000.d01-47.x

[40] Liu J, Clough SJ, Hutchinson AJ, Adamah-Biassi EB, Popovska-Gorevski M, Dubocovich ML. MT1 and MT2 Melatonin Receptors: A Therapeutic Perspective. Annu Rev Pharmacol Toxicol. 2016; 56:361–83. 10.1146/annurev-pharmtox-010814-124742 26514204 PMC5091650

[41] Jumnongprakhon P, Sivasinprasasn S, Govitrapong P, Tocharus C, Tocharus J. Activation of melatonin receptor (MT1/2) promotes P-gp transporter in methamphetamine-induced toxicity on primary rat brain microvascular endothelial cells. Toxicol In Vitro. 2017; 41:42–48. 10.1016/j.tiv.2017.02.010 28223141

[42] Xia Y, Chen S, Zeng S, Zhao Y, Zhu C, Deng B, Zhu G, Yin Y, Wang W, Hardeland R, Ren W. Melatonin in macrophage biology: Current understanding and future perspectives. J Pineal Res. 2019; 66:e12547. 10.1111/jpi.12547 30597604

[43] Jia P, Liu C, Wu N, Jia D, Sun Y. Agomelatine protects against myocardial ischemia-reperfusion injury by inhibiting mitochondrial permeability transition pore opening. Am J Transl Res. 2018; 10:1310–23. 29887947 PMC5992559

[44] Papp M, Gruca P, Boyer PA, Mocaër E. Effect of Agomelatine in the Chronic Mild Stress Model of Depression in the Rat. Neuropsychopharmacology. 2003; 28:694–703. 10.1038/sj.npp.1300091 12655314

[45] Martin V, Allaïli N, Euvrard M, Marday T, Riffaud A, Franc B, Mocaër E, Gabriel C, Fossati P, Lehericy S, Lanfumey L. Effect of agomelatine on memory deficits and hippocampal gene expression induced by chronic social defeat stress in mice. Sci Rep. 2017; 8:45907. 10.1038/srep45907 28374847 PMC5379201

[46] Kusaka I, Kusaka G, Zhou C, Ishikawa M, Nanda A, Granger DN, Zhang JH, Tang J. Role of AT1 receptors and NAD(P)H oxidase in diabetes- aggravated ischemic brain injury. Am J Physiol Heart Circ Physiol. 2004; 286:H2442–51. 10.1152/ajpheart.01169.2003 15148062

[47] Wang P, Xu TY, Guan YF, Tian WW, Viollet B, Rui YC, Zhai QW, Su DF, Miao CY. Nicotinamide phosphoribosyltransferase protects against ischemic stroke through SIRT1-dependent adenosine monophosphate-activated kinase pathway. Ann Neurol. 2011; 69:360–74. 10.1002/ana.22236 21246601

